# Hemodialysis reinitiation using a resurrected mummy fistula: a case report

**DOI:** 10.1186/s12882-018-1089-9

**Published:** 2018-10-26

**Authors:** Ziming Wan, Qiquan Lai, Bo Tu

**Affiliations:** 1 Department of Nephrology, The First Affiliated Hospital of Chongqing Medical University, 1 Youyi Road, Chongqing, 400016 China; 2 Department of Ultrasonography, The First Affiliated Hospital of Chongqing Medical University, 1 Youyi Road, Chongqing, 400016 China

**Keywords:** Arteriovenous fistula, Occlusion, Revascularization, Ultrasound

## Abstract

**Background:**

Kidney allograft loss becomes an important cause of end-stage kidney disease and requires dialysis reinitiation. We report a case of a patient who restarted hemodialysis after his second kidney graft failure using a long-discarded autologous arteriovenous fistula.

**Case presentation:**

A 62-year-old man was diagnosed with end-stage renal disease 20 year ago, and a native arteriovenous fistula was created for hemodialysis. After the patient received his first kidney transplantation, the hemodialysis fistula was discarded and chronically thrombosed for 13 years. When the patient experienced his second kidney graft loss and presented with uremia again, dialysis restart was needed. Under vascular ultrasound, but not x-ray, guidance, we successfully revascularized the patient’s chronically occluded, long-discarded arteriovenous fistula access and used it for hemodialysis. The resurrected fistula remained patent and clinically useable for hemodialysis up to 18 months.

**Conclusions:**

This report provides the feasibility of ultrasound-guided transluminal angioplasty for the treatment of a mummy hemodialysis fistula, which could be considered when managing patients who need dialysis reinitiation.

**Electronic supplementary material:**

The online version of this article (10.1186/s12882-018-1089-9) contains supplementary material, which is available to authorized users.

## Background

Kidney allograft loss has become one of the leading causes of kidney failure which requires dialysis [1, 2]. Patients with failed kidney transplant have greater mortality than transplant-naive patients [3]. Although timing of dialysis reinitiation in failed transplant patients is controversial, patients with uremic signs and symptoms usually need to start hemodialysis immediately [1, 2, 4]. Autologous arteriovenous fistula (AVF) has been the gold standard of hemodialysis vascular access for decades. However, limited data are available regarding the choice of vascular access for dialysis reinitiation. Typically, the vascular access, which was created and used prior to kidney transplantation, has been discarded and chronically occluded when patients need re-start hemodialysis after graft loss. Newly created AVFs require time to mature before being clinically usable. In recent years, endovascular strategies have been developed to treat stenotic or thrombosed vascular access [5, 6]. For instance, percutaneous transluminal angioplasty has been used to restore acutely or recently occluded hemodialysis fistulas, which was proved to be superior than open surgical intervention [7, 8]. However, revascularization of chronically occluded fistulas with percutaneous angioplasty is challenging [9]. To avoid toxic effects of contrast media on failing kidneys, x-ray-free ultrasound-guided endovascular techniques were introduced to treat stenosis of AVFs [7, 10–12]. So far, ultrasound-guided percutaneous angioplasty has not yet been applied to revascularization of AVF chronic total occlusion. We describe a patient who experienced a second kidney allograft loss and needed hemodialysis reinitiation, but his native AVF had been abandoned and thrombosed for as long as 13 years. Here we share the experience of successful ultrasound-guided revascularization of the chronically occluded hemodialysis fistula.

## Case presentation

A 62-year-old man was diagnosed with end-stage renal disease 20 year ago, and a native AVF was created at the left wrist for hemodialysis treatments. One year later, the patient received his first kidney transplant in the right iliac fossa and took a combination of immunosuppressive medications (including azathioprine, cyclosporin A, and corticosteroids). After that, the AVF was abandoned. Fifteen years ago, physical examination found that the AVF was occluded. At 6 years after renal transplantation, the patient suffered from lower extremity edema and was diagnosed with acute kidney transplant rejection based on renal biopsy. The failed transplanted kidney was surgically removed, and the patient received his second kidney transplant in the left iliac fossa. After the transplantation, the patient continued immunosuppressive therapy, with serum creatinine levels ranging between 120 and 130 μmol/L.

**Fig. 1:**
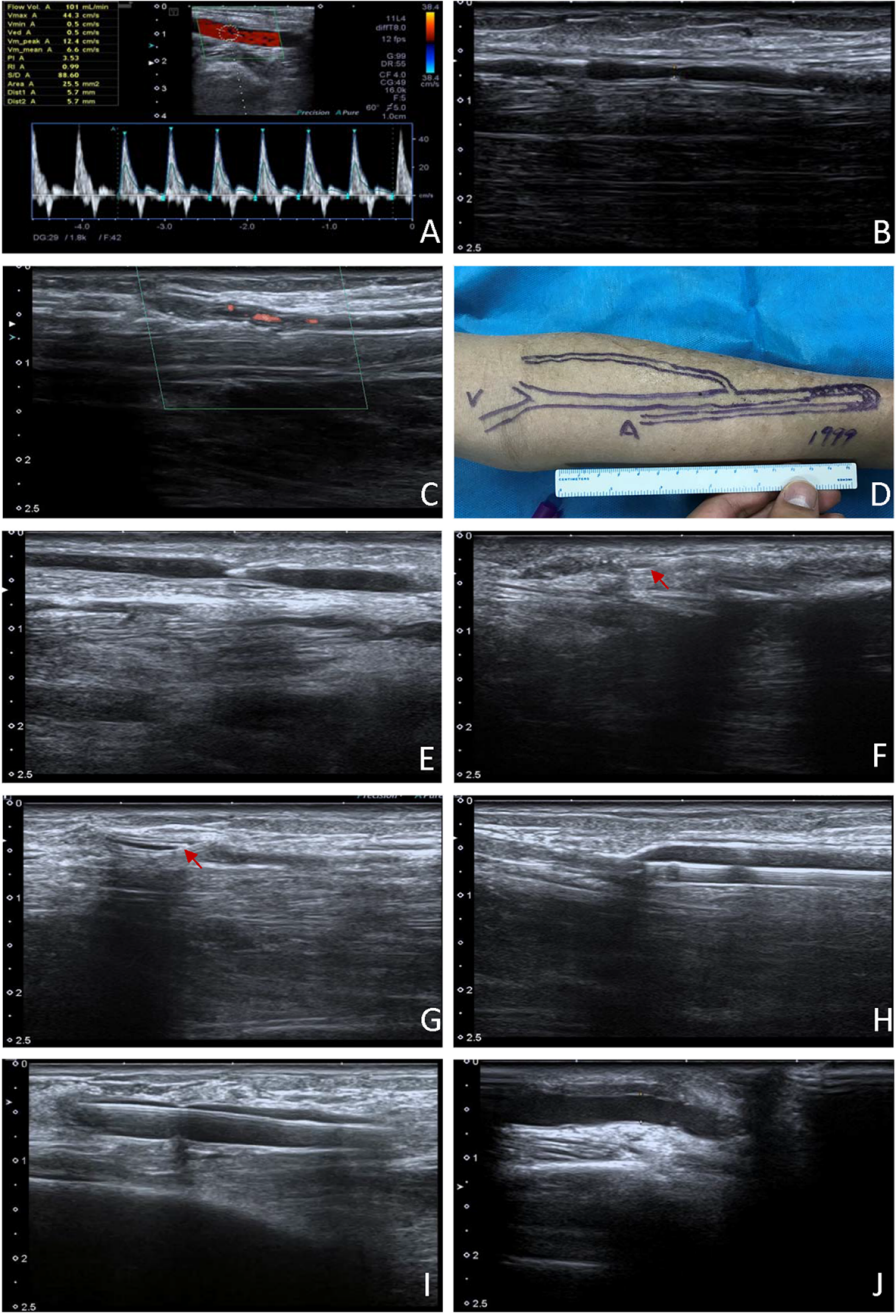
Ultrasound-guided percutaneous angioplasty of the chronically occluded arteriovenous fistula (AVF) for hemodialysis. **a** Blood flow in the brachial artery. **b** Ultrasound image of the radial artery. **c** No blood flow in the totally occluded AVF before intervention. **d** Schematic of the occluded AVF. **e** A 6-Fr sheath was retrogradely inserted into the fistulous vein. **f** A guidewire failed to cross the chronic total occlusion lesion. Arrow shows the curved tip of the wire. **g** The guidewire crossed the lesions with the support of a 6-Fr angiographic catheter. Arrow shows the tip of the wire. The radial artery (**h)** and AVF (**i**) was dilated with a balloon. **j** The AVF after balloon dilation

Eighteen months ago, the patient presented with orthopnea, nausea, and vomiting, and was admitted to the Department of Nephrology. On physical examination, there was no thrill or pulse over the AVF and no bruit on auscultation. Vascular ultrasound examination revealed the patent brachial (Fig. 1a, blood flow: 101 mL/min) and radial (Fig. 1b, diameter: 1.1 mm) arteries and the totally occluded AVF with no blood flow (Fig. 1c), which was illustrated in the skin (Fig. 1d). Serum creatinine concentration was 853 μmol/L and blood urea nitrogen was 34.1 mmol/L. The patient was diagnosed with renal allograft failure, and immediate hemodialysis restart was required. To promptly prepare a vascular access, we decided to attempt percutaneous revascularization of the patient’s chronically occluded AVF. Since we already had successful experience in treating stenotic and acutely thrombosed AVFs with ultrasound-guided transluminal angioplasty, we performed the revascularization under ultrasound using the Apollo 500 system (Toshiba, Tokyo, Japan), equipped with a 9–18 MHz linear transducer probe. The AVF was retrogradely punctured with a 22-G needle, and a 6-Fr sheath (Terumo, Tokyo, Japan) was retrogradely inserted into the fistulous vein (Fig. 1e). At first, a 0.014-in. Hi-Torque BMW Elite guidewire (Abbott Vascular, Santa Clara, CA, USA) was advanced to the lesion, but failed to cross the chronic total occlusion lesion at the AVF after switching the wire tip direction several times (Fig. 1f). An additional movie file shows this in more detail (see Additional file 1). With the support of a 6-Fr angiographic catheter advanced to the occlusion site, the guidewire was carefully and gradually inserted the chronic total occlusion lesion and successfully crossed the lesions at the fistula, anastomosis, and the radial artery, and parked in the radial artery (Fig. 1g). An additional movie file shows this in more detail (see Additional file 2). Subsequently, the radial artery was dilated with a 4.0 mm × 20 mm TREK balloon (Abbott Vascular) three times at 4, 8, and 12 atm (Fig. 1h), and the occluded lesion was dilated at 12, 18, and 20 atm. Then, further dilation of the lesion was performed with a 6.0 mm × 40 mm TREK balloon (Abbott Vascular) three times at 12 and 20 atm (Fig. 1i). The procedure took 54 min. After the dilation, the diameter and blood flow of the radial artery were 3.3 mm and 138 cm/s, respectively; and the cephalic vein diameter and blood flow were 2.9 mm and 251 cm/s, respectively. The diameter of the anastomose site was 3.2 mm (Fig. 1j).

**Fig. 2:**
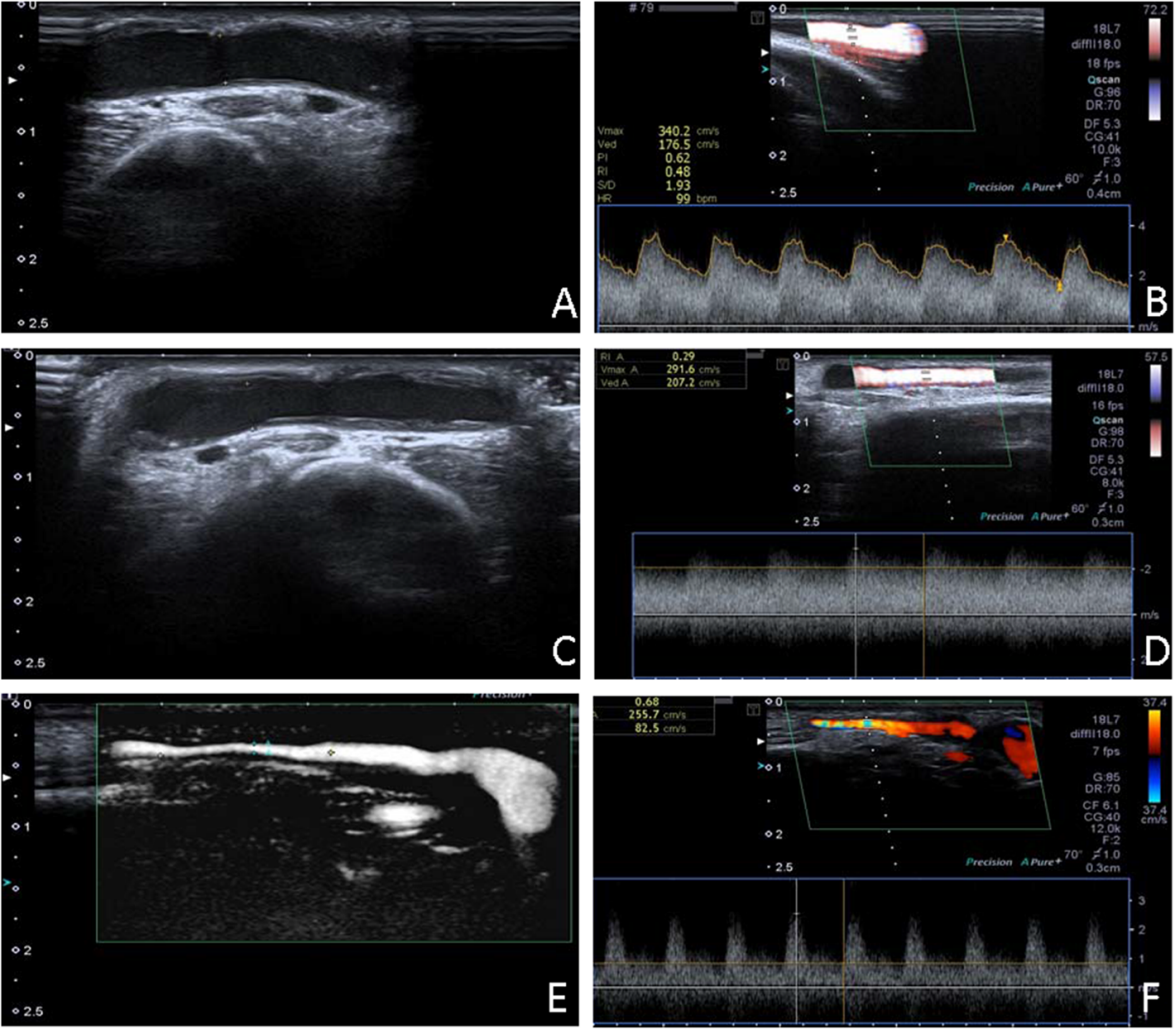
Vascular ultrasound follow-up after the endovascular treatment. At the 2-, 6-, and 18-month follow-ups, the anastomose site diameter and AVF blood flow were 3.8 mm (**a**) and 340 cm/s (**b**), 3.8 mm (**c**) and 291 cm/s (**d**), and 0.8 mm (**e**) and 255 cm/s (**f**), respectively

The revascularized AVF was used for hemodialysis after the intervention and remains patent for as long as 18 months. At the 2-, 6-, and 18-month follow-ups, the anastomose site diameter and AVF blood flow were 3.8 mm and 340 cm/s (Fig. 2a and b), 3.8 mm and 291 cm/s (Fig. 2c and d), and 0.8 mm and 255 cm/s (Fig. 2e and f), respectively. Although the anastomose site was stenotic at 18-month visit, the proximal AVF segment was normal with a diameter of 4.5 mm.

## Discussion and conclusions

Although percutaneous angioplasty has been widely used in managing patients with stenosis or acute thrombosis of hemodialysis AVFs [13], endovascular treatment for chronic total occlusion was rarely reported. In 2011, Pan et al. reported a series of 15 patients with chronically thrombosed hemodialysis fistulas and grafts which were resurrected with endovascular balloon angioplasty under x-ray guidance [9]. The vascular access of those patients had been discarded for up to 7 years. The age of our patient’s vascular access had been abandoned and occluded for 13 years; and this is so far the longest-discarded mummy AVF which had been successfully revascularized by endovascular angioplasty.

Since hemodialysis vascular access is superficial, they are easily accessible by ultrasonography. The advantages of ultrasound-guided over x-ray-guided fistula angioplasty include that there is neither toxic effects of contrast media on failing kidneys nor x-ray exposure [14]. Ultrasound-guided angioplasty has been used to rescue stenotic and newly thrombosed fistulas [15], but it is much more challenging to salvage chronically thrombosed hemodialysis fistulas. Vessel segments with total occlusion lesions are invisible under fluoroscopy but are detectable under ultrasound based on distinct echo intensity of the vessel wall and the luminal lesions. This feature enables ultrasound to be used for revascularization of totally occluded superficial vessels. In this report, we described a miracle of successful revascularization of a mummy fistula which was initially created for hemodialysis before the patient underwent his first kidney transplantation and was re-used 17 years later when he needed dialysis reinitiation after the loss of his second kidney allograft. This report provides an example of the feasibility of ultrasound-guided percutaneous balloon angioplasty for the treatment of long-discarded hemodialysis fistula, which could be considered when managing patients who require dialysis reinitiation.

Taken together, long-discarded native AVF could be a choice for hemodialysis reinitiation in patients with failed kidney transplant. Ultrasound-guided transluminal angioplasty is a feasible treatment for revascularization of chronically occluded AVF.

## Additional files

Additional file 1: A video shows attempts to pass the occluded lesion with a wire. (MP4 28153 kb)

Additional file 2: A video shows that the wire successfully passed the occluded lesion with the support of a catheter. (MP4 38645 kb)

## Abbreviation

AVF: Arteriovenous fistula
